# Cumulant-Based DOA Estimation of Noncircular Signals against Unknown Mutual Coupling

**DOI:** 10.3390/s20030878

**Published:** 2020-02-06

**Authors:** Baoping Wang, Junhao Zheng

**Affiliations:** 1 National Key Laboratory of Science and Technology on UAV, Northwestern Polytechnical University, Xi’an 710072, China; 2 School of Electronics and Information, Northwestern Polytechnical University, Xi’an 710072, China; zjh1071218679@163.com

**Keywords:** fourth-order cumulants (FOC), non-circularity, mutual coupling, rank-reduction (RARE)

## Abstract

To effectively find the direction of non-circular signals received by a uniform linear array (ULA) in the presence of non-negligible perturbations between array elements, i.e., mutual coupling, in colored noise, a direction of arrival (DOA) estimation approach in the context of high order statistics is proposed in this correspondence. Exploiting the non-circularity hidden behind a certain class of wireless communication signals to build up an augmented cumulant matrix, and carrying out a reformulation of the distorted steering vector to extract the angular information from the unknown mutual coupling, by exploiting the characteristic of mutual coupling, i.e., a limited operating range and an inverse relation of coupling effects to interspace, we develop a MUSIC-like estimator based on the rank-reduction (RARE) technique to directly determine directions of incident signals without mutual coupling compensation. Besides, we provide a solution to the problem of coherency between signals and mutual coupling between sensors co-existing, by selecting a middle sub-array to mitigate the undesirable effects and exploiting the rotation-invariant property to blindly separate the coherent signals into different groups to enhance the degrees of freedom. Compared with the existing robust DOA methods to the unknown mutual coupling under the framework of fourth-order cumulants (FOC), our work takes advantage of the larger virtual array and is able to resolve more signals due to greater degrees of freedom. Additionally, as the effective aperture is virtually extended, the developed estimator can achieve better performance under scenarios with high degree of mutual coupling between two sensors. Simulation results demonstrate the validity and efficiency of the proposed method.

## 1. Introduction

Direction of arrival (DOA) estimation, an important research area of sensor array signal processing, has attracted a large amount of attention because of its wide applications to electromagnetic, acoustic, seismic sensing, etc [1,2,3,4,5,6]. However, non-negligible interaction between sensors, i.e., mutual coupling, makes prevailing DOA estimation algorithms invalid since extra unknowns are introduced. Weiss and Friedlander [7] first discuss the structure of the mutual coupling matrix (MCM) in uniform linear and circular arrays and estimated the DOAs and mutual coupling coefficients in an iterative way. This work is followed by Sellone et al. whose approach is more robust to the detrimental effects and does not need a preliminary estimate [8]. Both schemes suffer from high computational complexity, which is inevitable given their reliance on multidimensional search processes. To circumvent the adverse effects of mutual coupling, the technique in [9,10] uses a subset of auxiliary sensors to make the “middle sub-array” mutual coupling free, and prevalent super-resolution algorithms can be directly applied the middle sub-array to resolve the DOAs. The main merit of these kind methods is the lower computational complexity due to not requiring any iterations but the degrees of freedom (DOFs) is reduced since only the middle sub-array is utilized. In a departure from middle sub-array methods [9,10], a recently proposed subspace-based method [11] takes advantage of the whole array by reparameterizing the actual array response and, hence improves the estimation accuracy. Recently, some novel sparsity inducing algorithms are developed to have robustness to the electromagnetic nuisance. Liu and Zhou first deal with the issue via the perspective of sparse Bayesian learning [12]. $\ell_{1}$-SVD is applied in [13] in conjunction with transforming the steering vector with mutual coupling, which is favorable for sparse recovery. Chen et al. investigate the problem of off-grid DOA estimation with coupling effects, and adopt the $\ell_{p}$-norm-based technique [14] and the relevance vector machine [15], respectively, to handle different cases, reducing amount of computation while preserving satisfactory estimation performance. Taking advantage of the uncorrelation between signals, Wang et al. address the issue from the perspective of group sparsity reconstruction of a long vector with mitigated noise components, and bring about an even better estimation accuracy and resolution [16].

The aforementioned work provides the solutions following the second-order statistics (SOS) and is unable to work properly in spatial colored noise if the noise covariance matrix is not available in advance. Since high-order statistics are insensitive to colored noise and can inherently enhance the DOFs [17,18,19], some study has been devoted to the suppression of unknown mutual coupling and colored noise using fourth-order cumulants (FOC) [20,21,22,23,24]. However, there are many additional pseudo-estimates in [20] which may affect the estimation performance, especially for the case where the true DOAs do not lie on the pseudo-peaks, while only the middle sub-array is utilized in [21] reducing the array aperture. Though promising results are shown in [22] and its follow up work [23,24], the array aperture in the former is fixed to a part of the physical aperture while the extended aperture is presented in the latter but still constraint to the middle sub-array, which gives rise to incomplete analysis and mining of statistical information implicated in the higher-order moments.

Recently, non-circularity embedded into wireless communication signals has been exploited to enhance DOFs for DOA estimation in the context of second-order statistics [25,26,27,28,29,30,31,32,33,34,35,36,37]. However, little work has been done on leveraging non-circularity in high-order moments to combat the unknown mutual coupling. To fill this gap, in this correspondence, a new FOC-based approach is proposed for the direction finding of non-circular signals in the presence of the deleterious effects between sensors. Making use of the characteristic of mutual coupling, such as a limited operating range and an inverse relation of coupling effects to interspace, to parameterize the steering vector, as well as the non-circularity of the observations to form a virtual array, we develop a MUSIC-like estimator by means of the rank-reduction (RARE) property to resolve the DOA estimates without any calibration process. Additionally, we introduce a solution to the problem of coherent signal estimation in the presence of mutual coupling between sensors, mitigating the detrimental effects via a middle sub-array and enhancing the DOFs through blind separation of the coherent signals into different groups. Compared with the existing FOC-based methods [21,22,23,24], in our proposed method, the DOFs as well as the effective aperture are further improved and hence, better accuracy and resolution can be obtained. Additionally, we discuss the identifiability of DOA estimation which has not been done in previous work, and the theoretical analysis shows that the work in this submission has an essential advantage in identifying more sources compared with [21,22,23,24] if the array has a relatively strong coupling between sensors.

The rest of the paper is organized as follows. In Section 2, the array model is discussed, when non-circular data observed by a sensor array with mutual coupling is constructed. In Section 3, we first develop a MUSIC-like method to directly determine the DOA estimates in conjunction with the non-circularity, but also isolate the mutual coupling effects, then discuss an even more challenging scenario where non-circular signals from one user in the uplink channel are coherent to each other, provide a solution to separate the coherent signals into different groups with the help of the middle array that is coupling-free, and then perform a DOA estimation in each coherent group via the forward–backward spatial smoothing (FBSS) technique. The estimation performance in terms of identifiability and computational complexity is analyzed in Section 4. Simulation results in Section 6 show the significant improvement of the proposed method. Finally, some concluding remarks are given in Section 7.

Throughout this submission, the following notations will be adopted: the operators ${\left(\cdot \right)}^{T}$, ${\left(\cdot \right)}^{*}$, ${\left(\cdot \right)}^{H}$, ${\left(\cdot \right)}^{+}$, |·|, E[·], det{·}, ⊗, ∘, and ${∥\cdot ∥}_{2}$ denote the operation of transpose, conjugate, conjugate transpose, pseudo-inverse, modulus, expectation, determinant, Kronecker product, Khatri–Rao (KR) product, and Euclidean ($\ell_{2}$) norm, respectively. The symbol $\mathrm{diag}\{z_{1},\cdots ,z_{N}\}$ represents a diagonal matrix with diagonal entries $z_{1},\cdots ,z_{N}$, $\mathrm{blkdiag}\{Z_{1},Z_{2}\}$ symbolizes a block diagonal matrix with diagonal entries $Z_{1}$ and $Z_{2}$, $\mathrm{Toeplitz}\left\{\cdot \right\}$ represents a symmetric Toeplitz matrix constructed by a vector in the brace, and $\mathrm{cum}\{z_{1},z_{2},z_{3},z_{4}\}$ symbolizes a cumulant calculated from data $z_{1}$, $z_{2}$, $z_{3}$, and $z_{4}$. The symbol $I_{K}$ stands for an identity matrix of size K×K. The symbol Z(a:b,c:d) refers to a constructed sub-matrix by the entries from *a* to *b*-th row and *c* to *d*-th column of Z, and the symbol Z(a,b) denotes the entry in the *a*-th row and *b*-th column of Z.

## 2. Problem Formulation

### 2.1. Strictly Second-Order Non-Circular Signals

For a signal s(t), if its elliptic covariance is $E\left[s^{2}\left(t\right)\right]=\rho e^{j\phi }{E[|s\left(t\right)|}^{2}]=\rho e^{j\phi }\sigma_{s}^{2}\neq 0$, where ϕ is the deterministic non-circularity phase, and ρ is non-circularity rate satisfying 0<ρ≤1, then we say s(t) is non-circular. The special case ρ=1 is common in wireless communications, and the received baseband signals satisfying such a condition is referred to as strictly second-order non-circular, like amplitude modulation (AM), binary phase shift keying (BPSK), amplitude shift keying (ASK), offset quadrature phase shift keying (OQPSK), and pulse amplitude modulation (PAM), etc. [25,26,27,28,30,38,39]. To aid the understanding of such a particular class of signals, we take the baseband signal of BPSK or ASK modulation as an example. In the framework of DOA estimation, the analog received BPSK or ASK modulated signals are bandpass filtered and down converted to baseband, the in-phase and quadrature components are matched-filtered, sampled, and paired to obtain complex signal s(t) that can be factorized into $s\left(t\right)=e^{j\varphi }s_{0}\left(t\right)$ where $s_{0}\left(t\right)$ is a real-valued symbol and φ is an arbitrary phase shift, due to the initial phase of the transmitted carrier, that can be different for each signal but constant with time. Therefore, the baseband signal s(t) at the receiver side is complex-valued. This model has been used by numerous authors who have studied DOA estimation of non-circular signals [25,26,27,28,30,38,39]. Examining the covariance and elliptic covariance of s(t), one gets $E\left[s\left(t\right)s^{*}\left(t\right)\right]=E\left[e^{j\varphi }s_{0}\left(t\right)e^{-j\varphi }s_{0}\left(t\right)\right]=E\left[s_{0}^{2}\left(t\right)\right]=\sigma_{s}^{2}$ and $E\left[s(t)s(t)\right]=E\left[e^{j\varphi }s_{0}\left(t\right)e^{j\varphi }s_{0}\left(t\right)\right]=e^{j2\varphi }E\left[s_{0}^{2}\left(t\right)\right]=e^{j2\varphi }\sigma_{s}^{2}\neq 0$. By the definition of non-circular signal, one can readily find that ρ=1 and ϕ=2φ, i.e., the baseband signal of BPSK or ASK modulation is strictly non-circular, and the non-circularity phase is twice as much as the initial phase. For the case of the received baseband signals with unknown modulation, one can resort to two kinds of classification approaches to identify the modulation of the signals. One class is the likelihood-based approach [40,41,42] and the other is the so-called feature-based approach [43,44,45,46,47,48]. If the signals are recognized as non-circular, such as BPSK, ASK, OQPSK, or PAM, the proposed method in the sequel is applicable, otherwise it fails to work. The research on modulation classification is beyond the scope of this manuscript, so we assume that the modulation of the received baseband signals is known *a priori* or can be recognized by using existing classification approaches if not specified, and the signals to be dealt with are strictly second-order non-circular.

### 2.2. Array Model for Non-Circular Signals

Consider narrowband non-circular signals $s_{0,i}\left(t\right)$ in the far filed impinge on an *M*-elements uniform linear array (ULA) from *N* different angles $\theta_{i}$, i=1,2,⋯,N, and the mutual coupling between sensors cannot be neglected, then at the time index *t* the corresponding array observation can be expressed as

(1) $$\begin{array}{cc} x(t) & =\sum_{i=1}^{N}Ca\left(\theta_{i}\right)e^{j\frac{\phi_{i}}{2}}s_{0,i}\left(t\right)+n\left(t\right)=CA\Psi s_{0}\left(t\right)+n\left(t\right) \end{array}$$

where $a\left(\theta \right)={\left[\begin{array}{c} 1,\beta \left(\theta \right),\cdots ,\beta^{M-1}\left(\theta \right) \end{array}\right]}^{T}\in C^{M}$ is the steering vector, $\beta \left(\theta \right)=e^{j\frac{2\pi d}{\lambda }\sin\theta }$, λ, and *d* are the carrier wavelength and the spacing between adjacent sensors, respectively, C denotes the MCM quantifying the degrees of electromagnetic coupling among elements, $A=\left[\begin{array}{c} a\left(\theta_{1}\right),\cdots ,a\left(\theta_{N}\right) \end{array}\right]$ is the array manifold, $\Psi =\mathrm{diag}\left\{e^{j\frac{\phi_{1}}{2}},\cdots ,e^{j\frac{\phi_{N}}{2}}\right\}$, $s_{0}\left(t\right)={\left[\begin{array}{c} s_{0,1}\left(t\right),\cdots ,s_{0,N}\left(t\right) \end{array}\right]}^{T}\in R^{N}$, and n(t) is the noise following Gaussian distribution but spatially colored. In addition, it is assumed that A is unambiguous, i.e., the steering vectors ${\left\{a\left(\theta_{i}\right)\right\}}_{i=1}^{N}$ are linearly independent for any set of distinct ${\left\{\theta_{i}\right\}}_{i=1}^{N}$.

As described in [9,10,11,49,50,51], one can sufficiently model the perturbed ULA by taking the electromagnetic characteristic of mutual coupling, such as a limited operating range and an inverse relation of coupling effects to interspace, into consideration. To be specific, If the mutual coupling acting on two sensors covers *P* interelement spacing at most such that the resultant MCM has finite non-zero coefficients, then the resultant MCM is banded symmetric Toeplitz, which can be formulated as

(2) $$\begin{array}{cc} C & =\mathrm{Toeplitz}\left\{\left[1,c_{1},\cdots ,c_{P-1},0_{1\times (M-P)}\right]\right\} \end{array}$$

where $0<|c_{1}\left|,\right|c_{2}\left|,\cdots ,\right|c_{P-1}|<c_{0}=1$ are the mutual coupling coefficients.

Since the incident signals follow strictly second-order non-circularity, they are deterministic and non-Gaussian, and the FOC matrices from the observation blocks can be given by

(3) $$\begin{array}{cc} C_{x1} & =\mathrm{cum}\left\{x^{*}\left(t\right),x^{T}\left(t\right),x^{*}\left(t\right),x^{T}\left(t\right)\right\} \end{array}$$

(4) $$\begin{array}{cc} C_{x2} & =\mathrm{cum}\left\{x\left(t\right),x^{T}\left(t\right),x\left(t\right),x^{T}\left(t\right)\right\} \end{array}$$

whose entries in the $\left[\left(k_{1}-1\right)M+k_{2}\right]$-th row and the $\left[\left(l_{1}-1\right)M+l_{2}\right]$-th column, $k_{1},k_{2},l_{1},l_{2}=1,2,\cdots ,M$, are defined as

(5) $$\begin{array}{cc} \; & \;\;C_{x1}\left(\left(k_{1}-1\right)M+k_{2},\left(l_{1}-1\right)M+l_{2}\right)\\ \; & =\mathrm{cum}\{x_{k_{1}}^{*}\left(t\right),x_{l_{1}}\left(t\right),x_{k_{2}}^{*}\left(t\right),x_{l_{2}}\left(t\right)\}\\ \; & =E\left[x_{k_{1}}^{*}\left(t\right)x_{k_{2}}^{*}\left(t\right)x_{l_{1}}\left(t\right)x_{l_{2}}\left(t\right)\right]-E\left[x_{k_{1}}^{*}\left(t\right)x_{k_{2}}^{*}\left(t\right)\right]\\ \; & \;\times E\left[x_{l_{1}}\left(t\right)x_{l_{2}}\left(t\right)\right]-E\left[x_{k_{1}}^{*}\left(t\right)x_{l_{1}}\left(t\right)\right]E\left[x_{k_{2}}^{*}\left(t\right)x_{l_{2}}\left(t\right)\right]\\ \; & \;-E\left[x_{k_{1}}^{*}\left(t\right)x_{l_{2}}\left(t\right)\right]E\left[x_{k_{2}}^{*}\left(t\right)x_{l_{1}}\left(t\right)\right] \end{array}$$

(6) $$\begin{array}{cc} \; & \;\;C_{x2}\left(\left(k_{1}-1\right)M+k_{2},\left(l_{1}-1\right)M+l_{2}\right)\\ \; & =\mathrm{cum}\{x_{k_{1}}\left(t\right),x_{l_{1}}\left(t\right),x_{k_{2}}\left(t\right),x_{l_{2}}\left(t\right)\}\\ \; & =E\left[x_{k_{1}}\left(t\right)x_{k_{2}}\left(t\right)x_{l_{1}}\left(t\right)x_{l_{2}}\left(t\right)\right]-E\left[x_{k_{1}}\left(t\right)x_{k_{2}}\left(t\right)\right]\\ \; & \;\times E\left[x_{l_{1}}\left(t\right)x_{l_{2}}\left(t\right)\right]-E\left[x_{k_{1}}\left(t\right)x_{l_{1}}\left(t\right)\right]E\left[x_{k_{2}}\left(t\right)x_{l_{2}}\left(t\right)\right]\\ \; & \;-E\left[x_{k_{1}}\left(t\right)x_{l_{2}}\left(t\right)\right]E\left[x_{k_{2}}\left(t\right)x_{l_{1}}\left(t\right)\right] \end{array}$$

where $x_{m}\left(t\right)$ is the *m*-th entry of x(t). Substituting Equation (1) into Equations (3) and (4), and utilizing the properties of high-order statistics [CP1]–[CP5] in [18], one can proceed to

(7) $$\begin{array}{cc} C_{x1} & =\mathrm{cum}\left\{{\left(CA\Psi s_{0}\left(t\right)\right)}^{*},{\left(CA\Psi s_{0}\left(t\right)\right)}^{T},{\left(CA\Psi s_{0}\left(t\right)\right)}^{*},\right.\\ \; & \;\left.{\left(CA\Psi s_{0}\left(t\right)\right)}^{T}\right\}+\mathrm{cum}\left\{n^{*}\left(t\right),n^{T}\left(t\right),{\left(n(t)\right)}^{*},{\left(n(t)\right)}^{T}\right\}\\ \; & =\mathrm{cum}\left\{\sum_{p=1}^{N}{\left(Ca(\theta_{p})\right)}^{*}e^{-j\frac{\phi_{p}}{2}}s_{0,p}^{*}\left(t\right),\sum_{m=1}^{N}{\left(Ca(\theta_{m})\right)}^{T}\right.\\ \; & \;\left.\times e^{j\frac{\phi_{m}}{2}}s_{0,m}\left(t\right),\sum_{q=1}^{N}{\left(Ca(\theta_{q})\right)}^{*}e^{-j\frac{\phi_{q}}{2}}s_{0,q}^{*}\left(t\right),\right.\\ \; & \;\left.\sum_{n=1}^{N}{\left(Ca(\theta_{n})\right)}^{T}e^{j\frac{\phi_{n}}{2}}s_{0,n}\left(t\right)\right\}\\ \; & =\sum_{p=1}^{N}\sum_{q=1}^{N}\sum_{m=1}^{N}\sum_{n=1}^{N}{\left(e^{j\frac{\phi_{p}+\phi_{q}}{2}}\left(Ca(\theta_{p})\right)⊗\left(Ca(\theta_{q})\right)\right)}^{*}\left(e^{j\frac{\phi_{m}+\phi_{n}}{2}}\left(Ca(\theta_{m})\right)\right.\\ \; & \;{\left.⊗\left(Ca(\theta_{n})\right)\right)}^{T}\mathrm{cum}\left\{s_{0,p}^{*}\left(t\right),s_{0,m}\left(t\right),s_{0,q}^{*}\left(t\right),s_{0,n}\left(t\right)\right\}\\ \; & =\sum_{i=1}^{N}{\left(\left(Ca(\theta_{i})\right)⊗\left(Ca(\theta_{i})\right)\right)}^{*}{\left(\left(Ca(\theta_{i})\right)⊗\left(Ca(\theta_{i})\right)\right)}^{T}\\ \; & \;\mathrm{cum}\{s_{0,i}\left(t\right),s_{0,i}\left(t\right),s_{0,i}\left(t\right),s_{0,i}\left(t\right)\}\\ \; & =\sum_{i=1}^{N}\gamma_{i}{\left(\left(Ca(\theta_{i})\right)⊗Ca\left(\theta_{i}\right)\right)}^{*}{\left(\left(Ca(\theta_{i})\right)⊗Ca\left(\theta_{i}\right)\right)}^{T}\\ \; & ={\left(\left(CA\right)\circ \left(CA\right)\right)}^{*}C_{s}{\left(\left(CA\right)\circ \left(CA\right)\right)}^{T} \end{array}$$

(8) $$\begin{array}{cc} C_{x2} & =\mathrm{cum}\left\{\left(CA\Psi s_{0}\left(t\right)\right),{\left(CA\Psi s_{0}\left(t\right)\right)}^{T},\left(CA\Psi s_{0}\left(t\right)\right),\right.\\ \; & \;\left.{\left(CA\Psi s_{0}\left(t\right)\right)}^{T}\right\}+\mathrm{cum}\left\{n\left(t\right),n^{T}\left(t\right),\left(n(t)\right),{\left(n(t)\right)}^{T}\right\}\\ \; & =\mathrm{cum}\left\{\sum_{p=1}^{N}Ca\left(\theta_{p}\right)e^{j\frac{\phi_{p}}{2}}s_{0,p}\left(t\right),\sum_{m=1}^{N}{\left(Ca(\theta_{m})\right)}^{T}\right.\\ \; & \;\left.\times e^{j\frac{\phi_{m}}{2}}s_{0,m}\left(t\right),\sum_{q=1}^{N}Ca\left(\theta_{q}\right)e^{j\frac{\phi_{q}}{2}}s_{0,q}\left(t\right),\right.\\ \; & \;\left.\sum_{n=1}^{N}{\left(Ca(\theta_{n})\right)}^{T}e^{j\frac{\phi_{n}}{2}}s_{0,n}\left(t\right)\right\}\\ \; & =\sum_{p=1}^{N}\sum_{q=1}^{N}\sum_{m=1}^{N}\sum_{n=1}^{N}\left(e^{j\frac{\phi_{p}+\phi_{q}}{2}}\left(Ca(\theta_{p})\right)⊗\left(Ca(\theta_{q})\right)\right)\\ \; & \;\times {\left(e^{j\frac{\phi_{m}+\phi_{n}}{2}}\left(Ca(\theta_{m})\right)⊗\left(Ca(\theta_{n})\right)\right)}^{T}\mathrm{cum}\{s_{0,p}\left(t\right),\\ \; & \;s_{0,m}\left(t\right),s_{0,q}\left(t\right),s_{0,n}\left(t\right)\}\\ \; & =\sum_{i=1}^{N}e^{j2\phi_{i}}\left(\left(Ca(\theta_{i})\right)⊗\left(Ca(\theta_{i})\right)\right)\left(\left(Ca(\theta_{i})\right)\right.\\ \; & \;{\left.⊗\left(Ca(\theta_{i})\right)\right)}^{T}\mathrm{cum}\left\{s_{0,i}\left(t\right),s_{0,i}\left(t\right),s_{0,i}\left(t\right),s_{0,i}\left(t\right)\right\}\\ \; & =\sum_{i=1}^{N}e^{j2\phi_{i}}\gamma_{i}\left(\left(Ca(\theta_{i})\right)⊗\left(Ca(\theta_{i})\right)\right){\left(\left(Ca(\theta_{i})\right)⊗\left(Ca(\theta_{i})\right)\right)}^{T}\\ \; & =\left(\left(CA\right)\circ \left(CA\right)\right)\Psi^{2}C_{s}\Psi^{2}{\left(\left(CA\right)\circ \left(CA\right)\right)}^{T} \end{array}$$

where $\gamma_{i}≜cum\left\{s_{0,i}\left(t\right),s_{0,i}\left(t\right),s_{0,i}\left(t\right),s_{0,i}\left(t\right)\right\}$ and $C_{s}≜\mathrm{diag}\left\{\gamma_{1},\cdots ,\gamma_{N}\right\}\in R^{N\times N}$.

## 3. Proposed Non-Circular FOC-Based Estimator

To increase the effective aperture as well as the DOFs, one can design an augmented cumulant matrix, of size $2M^{2}\times 2M^{2}$, as

(9) $$\begin{array}{cc} C_{x} & =\left[\begin{array}{cc} C_{x1} & C_{x2}^{*}\\ C_{x2} & C_{x1}^{*} \end{array}\right]\\ \; & =\left[\begin{array}{c} {\left(\left(CA\right)\circ \left(CA\right)\right)}^{*}\Psi^{-2}\\ \left(\left(CA\right)\circ \left(CA\right)\right)\Psi^{2} \end{array}\right]C_{s}{\left[\begin{array}{c} {\left(\left(CA\right)\circ \left(CA\right)\right)}^{*}\Psi^{-2}\\ \left(\left(CA\right)\circ \left(CA\right)\right)\Psi^{2} \end{array}\right]}^{H}. \end{array}$$

### DOA Estimation Without Mutual Coupling Compensation

The augmented matrix $C_{x}$ has the following factorization through the singular value decomposition (SVD)

(10) $$\begin{array}{cc} C_{x} & =U\Sigma V^{H} \end{array}$$

where $\Sigma =\mathrm{diag}\left\{\lambda_{1},\cdots ,\lambda_{2M^{2}}\right\}$ is composed of $2M^{2}$ singular values that can be sorted in descending order as $\lambda_{1}\geq \cdots \geq \lambda_{N}>\lambda_{N+1}=\cdots =\lambda_{2M^{2}}=0$. The matrix $U_{s}≜U\left(:,1:N\right)$ collects the singular vectors corresponding to the *N* largest singular values, while $U_{n}≜U\left(:,N+1:2M^{2}\right)$ contains the rest of the singular vectors corresponding to the $2M^{2}-N$ zero singular values. It is known that $U_{s}$ spans the signal subspace, while its orthogonal space, namely the noise subspace, is spanned by $U_{n}$, thus one has

(11) $$\begin{array}{cc} \; & {∥{\left[\begin{array}{c} e^{-j\phi_{i}}{\left(\left(Ca(\theta_{i})\right)⊗\left(Ca(\theta_{i})\right)\right)}^{*}\\ e^{j\phi_{i}}\left(Ca(\theta_{i})\right)⊗\left(Ca(\theta_{i})\right) \end{array}\right]}^{H}U_{n}∥}_{2}^{2}=0,\;\mathrm{for}\;i=1,2,\cdots ,N. \end{array}$$

However, due to the unknown C one cannot directly obtain the DOA estimates of the desired non-circular signals via 1-D search for making Equation (11) hold. For the purpose of decoupling the angular information from the electromagnetic impact between sensors of the ULA, we reparameterize the distorted steering vector as [11]

(12) $$\begin{array}{cc} Ca(\theta ) & =T(\theta )\alpha \end{array}$$

where

(13) $$\begin{array}{cc} T(\theta ) & =\mathrm{blkdiag}\left\{T_{1},T_{2},T_{3}\right\}\in C^{M\times (2P-1)} \end{array}$$

(14) $$\begin{array}{cc} \alpha & ={\left[\begin{array}{c} \mu_{1},\cdots ,\mu_{P-1},\tau \left(\theta \right),\alpha_{1},\cdots ,\alpha_{P-1} \end{array}\right]}^{T}\in C^{2P-1} \end{array}$$

with

(15) $$\begin{array}{cc} T_{1} & =\mathrm{diag}\left\{1,\beta \left(\theta \right),\cdots ,\beta^{P-2}\left(\theta \right)\right\}\in C^{\left(P-1)\times P-1\right)} \end{array}$$

(16) $$\begin{array}{cc} T_{2} & ={\left[\beta^{P-1}\left(\theta \right),\cdots ,\beta^{M-P}\left(\theta \right)\right]}^{T}\in C^{M-2P+2} \end{array}$$

(17) $$\begin{array}{cc} T_{3} & =\mathrm{diag}\left\{\beta^{M-P+1}\left(\theta \right),\cdots ,\beta^{M-1}\left(\theta \right)\right\}\in C^{\left(P-1)\times P-1\right)} \end{array}$$

(18) $$\begin{array}{cc} \mu_{l} & =1+\sum_{k=1}^{P-1}c_{k}\beta^{k}\left(\theta \right)+\sum_{k=1}^{l-1}c_{k}\beta^{-k}\left(\theta \right) \end{array}$$

(19) $$\begin{array}{cc} \alpha_{l} & =1+\sum_{k=1}^{P-1}c_{k}\beta^{-k}\left(\theta \right)+\sum_{k=1}^{P-1-l}c_{k}\beta^{k}\left(\theta \right) \end{array}$$

and

(20) $$\begin{array}{cc} \tau (\theta ) & =1+\sum_{k=1}^{P-1}c_{k}\left(\beta^{k}\left(\theta \right)+\beta^{-k}\left(\theta \right)\right). \end{array}$$

It should be noted that τ(θ) in Equation (14) is generally not equal to zero, otherwise “blind angles” cannot be identified [11]. Substituting Equation (12) back to Equation (11), one has

(21) $$\begin{array}{cc} \; & \;{∥{\left[\begin{array}{c} e^{-j\phi_{i}}{\left(\left(T(\theta_{i})\alpha \right)⊗\left(T(\theta_{i})\alpha \right)\right)}^{*}\\ e^{j\phi_{i}}\left(T(\theta_{i})\alpha \right)⊗\left(T(\theta_{i})\alpha \right) \end{array}\right]}^{H}U_{n}∥}_{2}^{2}\\ \; & ={∥{\left[\begin{array}{c} e^{-j\phi_{i}}{\left(\left(T\left(\theta_{i}\right)⊗T\left(\theta_{i}\right)\right)\left(\alpha ⊗\alpha \right)\right)}^{*}\\ e^{j\phi_{i}}\left(T\left(\theta_{i}\right)⊗T\left(\theta_{i}\right)\right)\left(\alpha ⊗\alpha \right) \end{array}\right]}^{H}U_{n}∥}_{2}^{2}\\ \; & ={∥\upsilon^{H}{\bar{T}}^{H}\left(\theta_{i}\right)U_{n}∥}_{2}^{2}\\ \; & =\upsilon^{H}{\bar{T}}^{H}\left(\theta_{i}\right)U_{n}U_{n}^{H}\bar{T}\left(\theta_{i}\right)\upsilon \\ \; & =\upsilon^{H}M\left(\theta_{i}\right)\upsilon =0 \end{array}$$

where $\upsilon ≜{\left[e^{-j\phi_{i}}{\left(\alpha ⊗\alpha \right)}^{H},e^{j\phi_{i}}{\left(\alpha ⊗\alpha \right)}^{T}\right]}^{T}\in C^{2{\left(2P-1\right)}^{2}}$, $\bar{T}\left(\theta_{i}\right)≜\mathrm{blkdiag}\left\{{\left(T\left(\theta_{i}\right)⊗T\left(\theta_{i}\right)\right)}^{*},T\left(\theta_{i}\right)⊗T\left(\theta_{i}\right)\right\}\in C^{2M^{2}\times 2{\left(2P-1\right)}^{2}}$, and $M\left(\theta_{i}\right)≜{\bar{T}}^{H}\left(\theta_{i}\right)U_{n}U_{n}^{H}\bar{T}\left(\theta_{i}\right)\in C^{2{\left(2P-1\right)}^{2}\times 2{\left(2P-1\right)}^{2}}$.

It is clear that ${\bar{T}}^{H}\left(\theta \right)U_{n}$ is of size $2{\left(2P-1\right)}^{2}\times \left(2M^{2}-N\right)$. If $2{\left(2P-1\right)}^{2}\leq 2M^{2}-N$, generally speaking, then the matrix ${\bar{T}}^{H}\left(\theta \right)U_{n}$ has a full row rank and M(θ) has a full rank accordingly. Nevertheless, for some special cases that θ is exactly the same with any one of the *N* true angles, i.e., $\theta =\theta_{i},i=1,\cdots ,N$, the expression in Equation (21) becomes zero. Since υ≠0, Equation (21) is valid only when M(θ) is singular that it takes as a zero-value determinant. As a result, the DOA estimation is now dependent on the determinant of M(θ). Since M(θ) does not contain any mutual coupling coefficients, $\det\left\{M(\theta )\right\}$ is insensitive to the nuisances. Therefore, one can estimate the bearings as

(22) $$\begin{array}{cc} \hat{\theta } & =\arg\min_{\theta }\det\left\{M(\theta )\right\}. \end{array}$$

## 4. Performance Analysis

In this section, the identifiability as well as the computational complexity of the proposed method is discussed, with a comparison of the existing work in the context of FOC, e.g., Liu’s [21] and Liao’s [24] approaches.

### 4.1. Identifiability of DOA Estimation

As $2{\left(2P-1\right)}^{2}\leq 2M^{2}-N$ makes M(θ) be of full rank, which is a necessary condition of the proposed algorithm, one can deduce that $N\leq 2M^{2}-2{\left(2P-1\right)}^{2}$, which implies that the maximum DOFs of the developed estimator are $2M^{2}-2{\left(2P-1\right)}^{2}$. By contrast, existing methods cannot estimate as many sources as our technique. Liao’s method is based on ESPRIT and as a result, the upper bound of estimation identifiability depends on the dimension of signal subspace. It is known that the signal subspace produced by Liao’s method has a size of M(M−2P+1)×N, and there are also (M−2P+2)(M−2P+1)−2(M−2P+1)=(M−2P)(M−2P+1) linearly dependent rows due to the property of the middle sub-array. Therefore, the row dimension of the signal subspace is 2P(M−2P+1), in other words, Liao’s method can estimate up to 2P(M−2P+1) signals. In Liu’s method, only the middle sub-array utilized, which means the extended aperture by FOC is 2(M−2P+2)−1 elements. Therefore, Liu’s method can handle at most 2(M−2P+1) DOAs. It is evident that the proposed method has the largest estimation capacity among the three algorithms, followed by Liao’s method and then Liu’s method. Consequently, Liu’s method suffers from the reduced effective aperture. Besides, if the coupling between sensors is prominent, i.e., *P* is large, or equivalently *M* is not sufficiently large, then the size of the middle sub-array will be very small. For instance, if $P=\frac{M}{2}$ and N=4, Liu’s method is not valid. This is because the effective array aperture [18] is 3 elements in this case, and at most 2 DOAs can be estimated.

### 4.2. Computational Complexity

Next, we compare the major computations of the proposed estimator, Liao’s and Liu’s methods, which involve in the fourth-order statistical matrices construction, SVD, and spectral search. For Liao’s approach, the main calculation cost is to form M−2P+2 FOC matrices of the same size M×M and to perform the SVD of the augmented M(M−2P+2)×M cumulant matrix, which requires $O\left(9\left(M-2P+2\right)M^{2}L+M^{3}\left(M-2P+2\right)\right)$ flops where *L* is the number of snapshots. Here, a flop stands for a complex-valued floating point multiplication operation. Liu’s technique constructs one ${\left(M-2P+2\right)}^{2}\times {\left(M-2P+2\right)}^{2}$ cumulant matrix, implements its SVD, and one-dimensional MUSIC spectral search, respectively, and it thus needs $O\left(9{\left(M-2P+2\right)}^{4}L+{\left(M-2P+2\right)}^{6}+\frac{180}{\delta }\left({\left(M-2P+2\right)}^{2}+1\right)\left({\left(M-2P+2\right)}^{2}-N\right)\right)$ flops in total where δ is the sampling grid spacing. By comparison, the proposed scheme establishes two $M^{2}\times M^{2}$ cumulant matrices, carries out SVDs of the augmented $2M^{2}\times 2M^{2}$ matrix, and executes a one-dimensional spectral search on determinants of a $2{\left(2P-1\right)}^{2}$ matrix. The resulting flops taken in our work are in order of $O\left(18M^{4}L+8M^{6}+\frac{1440}{\delta }{\left(2P-1\right)}^{6}\right)$. As in general *L* is relatively large, say several thousands, the proposed method has the highest computational complexity among the three algorithms, followed by Liao’s approach and then Liu’s technique.

## 5. Solution to the Case of Coherent Signals

It should be noted that the aforementioned method is applicable to incident signals being independent, and the coherency between signals may lead to severe performance degradation or even total failure due to the rank deficiency. This motivates us to provide a solution to the knotty problem, i.e., multiple groups of coherent signals received by a tightly coupled ULA in colored noise, and another direction finding algorithm based on FOC is proposed in this section, exploiting rotation-invariant property to blindly separate the coherent signals into different groups, and then resolving the DOAs in each coherent group via rank restoration techniques such as FBSS.

First, in order to mitigate the unknown mutual coupling, one can extract the observations of the middle sub-array by $\tilde{x}\left(t\right)=Wx\left(t\right)$ where the selection matrix $W=\left[\begin{array}{c} 0_{\left(M-2P+2)\times (P-1\right)},I_{M-2P+2},0_{\left(M-2P+2)\times (P-1\right)} \end{array}\right]$.

Consider that there are *K* groups of signals from *K* users sensed by the sensor array deployed at the base station where the *k*-th group has $P_{k}$ signals that are coherent to each other, but the signals from different groups are independent. Referring to [52,53,54], the array observation with a shrinking size can be rewritten as

(23) $$\begin{array}{cc} \bar{x}\left(t\right) & =\bar{A}\tilde{\Gamma }s\left(t\right)+\bar{n}\left(t\right) \end{array}$$

where $\bar{A}=JA$, $\tilde{\Gamma }=D\Gamma$ with $D=\mathrm{diag}\left\{\mu \left(\theta_{1}\right),\mu \left(\theta_{2}\right),\cdots ,\mu \left(\theta_{N}\right)\right\},\mu \left(\theta_{i}\right)=c_{P-1}+\cdots +\beta^{P-1}\left(\theta_{i}\right)+\cdots +c_{P-1}\beta^{2P-2}\left(\theta_{i}\right)$, and $\Gamma =\mathrm{blkdiag}\{\alpha_{1},\cdots ,\alpha_{K}\}$ with $\alpha_{k}={\left[\begin{array}{c} \alpha_{k1},\cdots ,\alpha_{kP_{k}} \end{array}\right]}^{T}$ collecting fading coefficients.

Let $\tilde{A}=\bar{A}\tilde{\Gamma }=\left[b_{1},\cdots ,b_{K}\right]$, being the so-called generalized array manifold, where $b_{k}=\left[a\left(\theta_{k1}\right),\cdots ,a\left(\theta_{kPk}\right)\right]{\tilde{\alpha }}_{k}$ is the generalized steering vector, one can get four cumulant matrices as follows

(24) $$\begin{array}{cc} \Xi_{1} & ≜\mathrm{cum}\left\{{\bar{x}}_{1}\left(t\right),{\bar{x}}_{1}^{*}\left(t\right),\bar{x}\left(t\right),{\bar{x}}^{H}\left(t\right)\right\}\\ \; & =\mathrm{cum}\left\{\sum_{m=1}^{K}\tilde{A}\left(1,m\right)e^{j\frac{\phi_{m}}{2}}s_{0,m}\left(t\right),\sum_{p=1}^{K}{\tilde{A}}^{*}\left(1,p\right)e^{-j\frac{\phi_{p}}{2}}s_{0,p}^{*}\left(t\right),\sum_{n=1}^{K}b_{n}e^{j\frac{\phi_{n}}{2}}s_{0,n}\left(t\right),\sum_{r=1}^{K}b_{r}^{H}e^{-j\frac{\phi_{r}}{2}}s_{0,r}^{*}\left(t\right)\right\}\\ \; & =\sum_{m=1}^{K}\sum_{n=1}^{K}\sum_{p=1}^{K}\sum_{r=1}^{K}e^{j\frac{\phi_{m}-\phi_{p}+\phi_{n}-\phi_{r}}{2}}\tilde{A}\left(1,m\right){\tilde{A}}^{*}\left(1,p\right)b_{n}b_{r}^{H}\mathrm{cum}\left\{s_{0,m}\left(t\right),s_{0,p}^{*}\left(t\right),s_{0,n}\left(t\right),s_{0,r}^{*}\left(t\right)\right\}\\ \; & =\sum_{i=1}^{K}{\left|\tilde{A}\left(1,i\right)\right|}^{2}b_{i}b_{i}^{H}\mathrm{cum}\left\{s_{0,i}\left(t\right),s_{0,i}^{*}\left(t\right),s_{0,i}\left(t\right),s_{0,i}^{*}\left(t\right)\right\}\\ \; & =\sum_{i=1}^{K}\gamma_{i}{\left|\tilde{A}\left(1,i\right)\right|}^{2}b_{i}b_{i}^{H}\\ \; & =\tilde{A}{\tilde{C}}_{s}{\tilde{A}}^{H} \end{array}$$

(25) $$\begin{array}{cc} \Xi_{2} & ≜\mathrm{cum}\left\{{\bar{x}}_{2}\left(t\right),{\bar{x}}_{1}^{*}\left(t\right),\bar{x(t)},{\bar{x}}^{H}\left(t\right)\right\}\\ \; & =\mathrm{cum}\left\{\sum_{m=1}^{K}\tilde{A}\left(2,m\right)e^{j\frac{\phi_{m}}{2}}s_{0,m}\left(t\right),\sum_{p=1}^{K}{\tilde{A}}^{*}\left(1,p\right)e^{-j\frac{\phi_{p}}{2}}s_{0,p}^{*}\left(t\right),\sum_{n=1}^{K}b_{n}e^{j\frac{\phi_{n}}{2}}s_{0,n}\left(t\right),\sum_{r=1}^{K}b_{r}^{H}e^{-j\frac{\phi_{r}}{2}}s_{0,r}^{*}\left(t\right)\right\}\\ \; & =\sum_{m=1}^{K}\sum_{n=1}^{K}\sum_{p=1}^{K}\sum_{r=1}^{K}e^{j\frac{\phi_{m}-\phi_{p}+\phi_{n}-\phi_{r}}{2}}\tilde{A}\left(2,m\right){\tilde{A}}^{*}\left(1,p\right)b_{n}b_{r}^{H}\mathrm{cum}\left\{s_{0,m}\left(t\right),s_{0,p}^{*}\left(t\right),s_{0,n}\left(t\right),s_{0,r}^{*}\left(t\right)\right\}\\ \; & =\sum_{i=1}^{K}\frac{\tilde{A}\left(2,m\right)}{\tilde{A}\left(1,m\right)}{\left|\tilde{A}\left(1,i\right)\right|}^{2}b_{i}b_{i}^{H}\mathrm{cum}\left\{s_{0,i}\left(t\right),s_{0,i}^{*}\left(t\right),s_{0,i}\left(t\right),s_{0,i}^{*}\left(t\right)\right\}\\ \; & =\sum_{i=1}^{K}\gamma_{i}\frac{\tilde{A}\left(2,m\right)}{\tilde{A}\left(1,m\right)}{\left|\tilde{A}\left(1,i\right)\right|}^{2}b_{i}b_{i}^{H}\\ \; & =\tilde{A}\tilde{D}{\tilde{C}}_{s}{\tilde{A}}^{H} \end{array}$$

(26) $$\begin{array}{cc} \Xi_{3} & ≜\mathrm{cum}\left\{{\bar{x}}_{1}\left(t\right),{\bar{x}}_{1}^{*}\left(t\right),\bar{x}\left(t\right),{\bar{x}}^{T}\left(t\right)\right\}\\ \; & =\mathrm{cum}\left\{\sum_{m=1}^{K}\tilde{A}\left(1,m\right)e^{j\frac{\phi_{m}}{2}}s_{0,m}\left(t\right),\sum_{p=1}^{K}{\tilde{A}}^{*}\left(1,p\right)e^{-j\frac{\phi_{p}}{2}}s_{0,p}\left(t\right),\sum_{n=1}^{K}b_{n}e^{j\frac{\phi_{n}}{2}}s_{0,n}\left(t\right),\sum_{r=1}^{K}b_{r}^{T}e^{j\frac{\phi_{r}}{2}}s_{0,r}\left(t\right)\right\}\\ \; & =\sum_{m=1}^{K}\sum_{n=1}^{K}\sum_{p=1}^{K}\sum_{r=1}^{K}e^{j\frac{\phi_{m}-\phi_{p}+\phi_{n}+\phi_{r}}{2}}\tilde{A}\left(1,m\right){\tilde{A}}^{*}\left(1,p\right)b_{n}b_{r}^{T}\mathrm{cum}\left\{s_{0,m}\left(t\right),s_{0,p}\left(t\right),s_{0,n}\left(t\right),s_{0,r}\left(t\right)\right\}\\ \; & =\sum_{i=1}^{K}e^{j\phi_{i}}{\left|\tilde{A}\left(1,i\right)\right|}^{2}b_{i}b_{i}^{T}\mathrm{cum}\left\{s_{0,i}\left(t\right),s_{0,i}\left(t\right),s_{0,i}\left(t\right),s_{0,i}\left(t\right)\right\}\\ \; & =\sum_{i=1}^{K}\gamma_{i}e^{j\phi_{i}}{\left|\tilde{A}\left(1,i\right)\right|}^{2}b_{i}b_{i}^{T}\\ \; & =\tilde{A}\Psi {\tilde{C}}_{s}\Psi {\tilde{A}}^{T} \end{array}$$

(27) $$\begin{array}{cc} \Xi_{4} & ≜\mathrm{cum}\left\{{\bar{x}}_{2}\left(t\right),{\bar{x}}_{1}^{*}\left(t\right),\bar{x}\left(t\right),{\bar{x}}^{T}\left(t\right)\right\}\\ \; & =\mathrm{cum}\left\{\sum_{m=1}^{K}\tilde{A}\left(2,m\right)e^{j\frac{\phi_{m}}{2}}s_{0,m}\left(t\right),\sum_{p=1}^{K}{\tilde{A}}^{*}\left(1,p\right)e^{-j\frac{\phi_{p}}{2}}s_{0,p}\left(t\right),\sum_{n=1}^{K}b_{n}e^{j\frac{\phi_{n}}{2}}s_{0,n}\left(t\right),\sum_{r=1}^{K}b_{r}^{T}e^{j\frac{\phi_{r}}{2}}s_{0,r}\left(t\right)\right\}\\ \; & =\sum_{m=1}^{K}\sum_{n=1}^{K}\sum_{p=1}^{K}\sum_{r=1}^{K}e^{j\frac{\phi_{m}-\phi_{p}+\phi_{n}+\phi_{r}}{2}}\tilde{A}\left(2,m\right){\tilde{A}}^{*}\left(1,p\right)b_{n}b_{r}^{T}\mathrm{cum}\left\{s_{0,m}\left(t\right),s_{0,p}\left(t\right),s_{0,n}\left(t\right),s_{0,r}\left(t\right)\right\}\\ \; & =\sum_{i=1}^{K}e^{j\phi_{i}}\frac{\tilde{A}\left(2,m\right)}{\tilde{A}\left(1,m\right)}{\left|\tilde{A}\left(1,i\right)\right|}^{2}b_{i}b_{i}^{T}\mathrm{cum}\left\{s_{0,i}\left(t\right),s_{0,i}\left(t\right),s_{0,i}\left(t\right),s_{0,i}\left(t\right)\right\}\\ \; & =\sum_{i=1}^{K}\gamma_{i}e^{j\phi_{i}}\frac{\tilde{A}\left(2,m\right)}{\tilde{A}\left(1,m\right)}{\left|\tilde{A}\left(1,i\right)\right|}^{2}b_{i}b_{i}^{T}\\ \; & =\tilde{A}\Psi \tilde{D}{\tilde{C}}_{s}\Psi {\tilde{A}}^{T} \end{array}$$

where ${\bar{x}}_{1}\left(t\right)$ and ${\bar{x}}_{2}\left(t\right)$ are the first and second columns of $\bar{x}\left(t\right)$, respectively.

Then, one can take the eigen-decomposition to $\Xi_{2}\Xi_{1}^{+}$ and $\Xi_{4}\Xi_{3}^{+}$ respectively to work out the scaled generalized steering vectors. To see how it works, one can perform the following operations:

(28) $$\begin{array}{cc} \Xi_{2}\Xi_{1}^{+}\tilde{A}\; & =\tilde{A}\tilde{D}{\tilde{C}}_{s}{\tilde{A}}^{H}{\left(\tilde{A}{\tilde{C}}_{s}{\tilde{A}}^{H}\right)}^{+}\tilde{A}\\ \; & =\tilde{A}\tilde{D}{\tilde{C}}_{s}{\tilde{A}}^{H}{\left({\tilde{A}}^{H}\right)}^{+}{\tilde{C}}_{s}^{-1}{\tilde{A}}^{+}\tilde{A}\\ \; & =\tilde{A}\tilde{D}{\tilde{C}}_{s}{\left({\tilde{A}}^{+}\tilde{A}\right)}^{H}{\tilde{C}}_{s}^{-1}\\ \; & =\tilde{A}\tilde{D} \end{array}$$

where in the third equality we have used the identity ${\tilde{A}}^{+}\tilde{A}=I_{K}$ since $\tilde{A}$ is of full column rank. Through eigen-decomposition, one has $\Xi_{2}\Xi_{1}^{+}={\tilde{E}}_{s}{\tilde{\Sigma }}_{s}{\tilde{E}}_{s}^{H}$, where ${\tilde{E}}_{s}$ contains the eigenvectors of $\Xi_{2}\Xi_{1}^{+}$ while ${\tilde{\Sigma }}_{s}$ is a diagonal matrix comprising eigenvalues. Equivalently, one can deduce that $\Xi_{2}\Xi_{1}^{+}{\tilde{E}}_{s}={\tilde{E}}_{s}{\tilde{\Sigma }}_{s}$. As a result, it is natural to reveal ${\tilde{E}}_{s}=\tilde{A}\Lambda$, where Λ is an arbitrary diagonal matrix with nonzero entries, and ${\tilde{\Sigma }}_{s}=\tilde{D}$. Following the same principle, one can also obtain the eigenvectors of $\Xi_{4}\Xi_{3}^{+}$ as the scaled generalized steering vectors. Denoting ${\tilde{u}}_{k}$ and ${\overset{˘}{u}}_{k}$ are the the eigenvectors of $\Xi_{2}\Xi_{1}^{+}$ and $\Xi_{4}\Xi_{3}^{+}$, respectively, one can get a more robust estimate of the scaled generalized steering vectors by averaging them, i.e.,

(29) $$\begin{array}{cc} {\tilde{b}}_{k} & ≜\beta b_{k}=\frac{1}{2}\left({\tilde{u}}_{k}+{\overset{˘}{u}}_{k}\right). \end{array}$$

Then one can perform FBSS to ${\tilde{b}}_{k}$ to recover the rank deficiency

(30) $$\begin{array}{cc} B_{k}^{fb} & =\frac{1}{2q}\sum_{i=1}^{q}\left[W_{i}{\tilde{b}}_{k}{\tilde{b}}_{k}^{H}W_{i}^{H}+J{\left(W_{i}{\tilde{b}}_{k}{\tilde{b}}_{k}^{H}W_{i}^{H}\right)}^{*}J\right] \end{array}$$

where $W_{i}=\left[0_{m\times (i-1)},I_{m},0_{m\times (q-i)}\right]$ is the selection matrix for the *i*-th sub-array, with m=M−2P+3−q being is the number of sensors in each sub-array, and $J\in R^{m\times m}$ is the exchange matrix. For the *k*-th group of coherent signals, one now can apply ESPRIT to $B_{k}^{fb}$ to determine the DOA estimates.

**Remark** **1.** *The parameter q plays a significant role in rank restoration since $rank\left(B_{k}^{fb}\right)$ after FBSS becomes 1+2q, and it also restricts the DOFs and effective aperture for estimation by m=M−2P+3−q. We consider two extreme cases to discuss the choices of q. If $q=\left\lceil \frac{P_{i}}{2}\right\rceil$, i=1,2⋯,K, i.e., q achieves its lower bound, then one barely restores the rank deficiency with the maximum number of sensors after FBSS, but this may cause not all coherent signals to be detected, especially at low signal-to-noise ratio (SNR) or for few snapshots. On the other hand, If $q=M-2P+2-P_{i}$, i.e., q achieves its upper bound, then one restores the rank deficiency with an excessive number of times of FBSS, but this may cause biased estimates due to only one dimensional noise subspace being available. To our empirical knowledge and simulation results, the proposed method performs well when q is selected appropriately between the two bounds. However, the optimum choice of q is still an open problem and no theoretical guidance for the selection has been provided. A plausible way to choose the “optimal” q is to check whether $B_{k}^{fb}$ induced by q has the largest discrepancy between the $P_{i}$ largest eigenvalues and the $m-P_{i}$ smallest eigenvalues, in which case $B_{k}^{fb}$ can be considered to have the largest SNR. Therefore, the optimal q can be obtained by finding $B_{k}^{fb}$ with the largest discrepancy between the signal and the noise subspaces. Although this method for choosing optimal q is completely ad-hoc, it seems to be very simple and effective from simulations.*

**Remark** **2.** *A cumulant requires many more computations than a covariance and, hence, the proposed FOC-based estimator of non-circular signals against mutual coupling works well on condition that the observation window is sufficiently long and the target is (quasi-) stationary. We attempted to implement the proposed method in a testbed composed of a Virtex-7 series FPGA and a TMS320C6x series DSP, but found that it is infeasible to complete the DOA estimation within a time less than the scale of milliseconds in the case of M=8 and L=2000 due to the cumbersome calculations of cumulants and the SVD of the augmented cumulant matrix $C_{x}$, of size 16×16. As a result, for some applications, like maneuverable targets, the coherence time of such cumulants is quite short, and it is hard and even unlikely to realize the proposed algorithm by the prevailing hardware available in the market. On the other hand, for some applications that expect not much real-time quality, such as radio, hydrological, or meteorological environment monitoring where the incident signals have cyclostationarity, cumulant-based signal selective algorithms can be implemented for location estimation of far-field signals; the price to be paid is the need for the large number of computations and large data lengths for reliable estimation of the cumulants.*

**Remark** **3.** *For the case of wideband signals, one can divide the observations at each channel into some (possibly overlapping) segments, where for each segment, a number of frequency sub-bands are computed by the short-time Fourier transform (STFT). The idea of frequency-domain processing is to decouple the wideband model into a multitude of narrowband models, and then at each frequency subband one can apply the proposed method to obtain M(f,θ) constructed by Equation (21). In the frequency-domain approach, the final step is to combine M(f,θ) at various frequencies to obtain a DOA spectrum fusion, that is, $\hat{\theta }=\arg\min_{\theta }\sum_{f\in B_{d}}\det\left\{M(\theta )\right\}$ where $B_{d}\subset \left[0,\frac{1}{2}\right]$ is the discrete normalized frequency band of the received signals. The combination in the spectrum fusion equation follows the principle of incoherent signal subspace method (ISSM) in [55]. By contrast, our solution is inapplicable to the focusing-based approaches [56,57], which is another category of DOA estimation algorithms for wideband signals, since initial DOA estimates required therein cannot be obtained by conventional beamforming in the presence of unknown mutual coupling.*

**Remark** **4.** *The array perturbations, such as mutual coupling, gain-phase errors, and sensor location uncertainties, can be calibrated by placing signal sources at known positions, which is the so-called active calibration [58,59,60,61,62,63,64,65]. However, the process of measuring the array manifold including various perturbations can be time consuming and expensive, and it is inconvenient or even infeasible to make the calibration source available in some cases. In addition to the deployment of signals of opportunity, keeping the calibration effective is another essential issue. Plenty of factors contribute to the variation of array response over time: gradual changes in the behaviors of the sensor itself, the electronic circuitry, and the analog-to-digital converter (due to thermal effects, aging of components, etc.), changes in the electromagnetic environment (e.g., metal objects beside an antenna array cause a distortion of the beam pattern), and changes in the sensor locations (e.g., an antenna array mounted on the vibrating wing of an aircraft or a hydrophone array towed behind a ship). The active calibration scheme has its inherent shortcomings that are difficult to be overcome and, hence, in this paper we resort to the self-calibration solution that is not reliant on the array manifold measurement as well as the calibration sources whose positions are known in advance.*

**Remark** **5.** *The received data can be prewhitened with a square-root of the covariance matrix of the colored noise, and then the covariance matrix of colored noise is reduced to an identity matrix accordingly, i.e., the colored noise becomes “isotropic”. It should be noted that this operation relies on a prerequisite that the noise covariance matrix is available in advance. Generally speaking, noise in the internal circuitry of the antenna elements is slowly time-varying, and the prewhitening technique works well if the colored noise is wide-sense stationary over a period of time. However, this prerequisite may not hold in some scenarios. For instance, in wireless communication systems, the cell sites equipped with antenna arrays always receive signals from users, so the noise covariance matrix cannot be obtained separately; the reverberation noise in sonar has a fast time-variance due to the underwater dynamic media, so the noise covariance matrix at the next moment varies dramatically from that at the previous moment. As a result, the prewhitening technique is invalid under these adverse circumstances while fourth-order cumulants can handle such challenges.*

## 6. Simulation Results and Discussion

In this section, numerous simulations are offered to assess the performance of the proposed non-circular FOC method that exploits the non-circular structure via actual steering vector reparameterization. Specifically, we compare our solution with its circular counterparts, Liu’s [21] and Liao’s approaches [24] that are based on the FOC. A ULA with half-wavelength spacing between adjacent elements is considered. Similar to the settings in [39], it is assumed that the BPSK modulated incident signals are statistically independent and have identical power. The noise is assumed to be spatially-colored Gaussian and the (m,n)-th entry of the covariance matrix is given by $R\left(m,n\right)=\sigma_{n}^{2}0.85^{\left|m-n\right|}e^{j\frac{\pi |m-n|}{16}}$ [66,67]. The signal-to-noise ratio (SNR) is defined as $10\log_{10}\left(\sigma_{s}^{2}/\sigma_{n}^{2}\right)$. The accuracy of the DOA estimate is measured from 1000 Monte Carlo runs in terms of the root mean square error (RMSE) which is defined as

(31) $$\begin{array}{c} \mathrm{RMSE}=\sqrt{\frac{1}{1000N}\sum_{n=1}^{1000}\sum_{i=1}^{N}{\left({\hat{\theta }}_{i}^{\left(n\right)}-\theta_{i}\right)}^{2}} \end{array}$$

where ${\hat{\theta }}_{i}^{\left(n\right)}$ is the estimate of $\theta_{i}$ for the *n*-th trial, and *N* is the number of signals.

**Figure 1:**
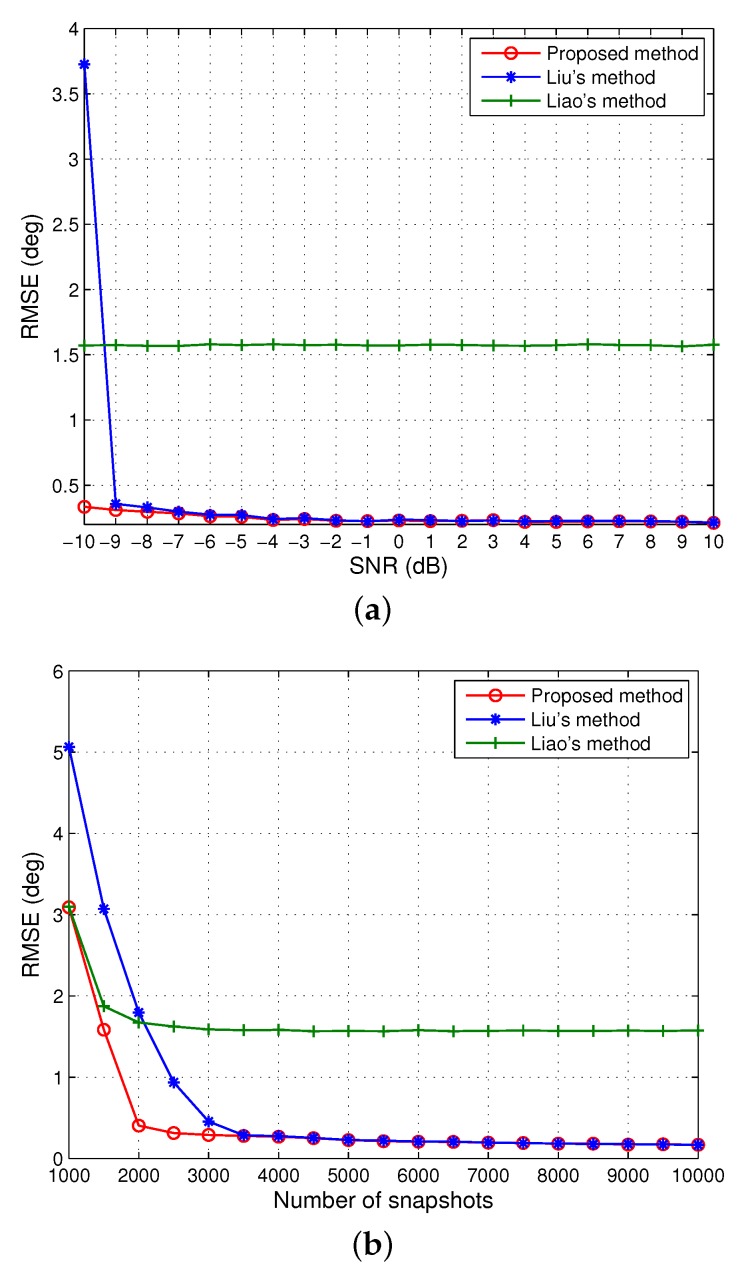
Root mean square error (RMSE) of the direction of arrival (DOA) estimates of four signals when M=10 and P=3. (**a**) The number of snapshots is 5000. (**b**) signal-to-noise ratio (SNR)=0 dB.

We first assumed that four independent sources from $\left[-23^{\circ },-6^{\circ },14^{\circ },36^{\circ }\right]$ impinging on a ten-element array with mutual coupling where P=3 with $c_{1}=-0.1545+0.4755j,c_{2}=0.1618-0.1176j$. From Figure 1, it can be seen that with the increase of the two variables, the SNR and the number of snapshots, the RMSE of the DOA estimates declines slowly for all three methods and then stabilizes for certain SNR or snapshot values due to the manifold error resulting from the unknown mutual coupling, whereas Liao’s method has a relatively large and constant error at approximately $1.57^{\circ }$. It is evident that our solution is superior to Liu’s method and Liao’s method, particularly at low SNRs as well as small snapshot sizes, since a larger array aperture has been utilized for in our algorithm. Between the other two approaches, one can tell that in this scenario, Liu’s method generally outperforms Liao’s method and achieves similar performance to our method in moderate conditions (i.e., SNR ≥−7 dB or the number of snapshots is larger than 3500) at a cost of computation. This demonstrates that when the ULA has sufficient sensors and the degree of coupling is not high (i.e., *P* is small), Liu’s method performs well. In contrast, Liao’s method has a clear advantage over Liu’s for SNR lower than −10 dB or for less than 2000 snapshots.

**Figure 2:**
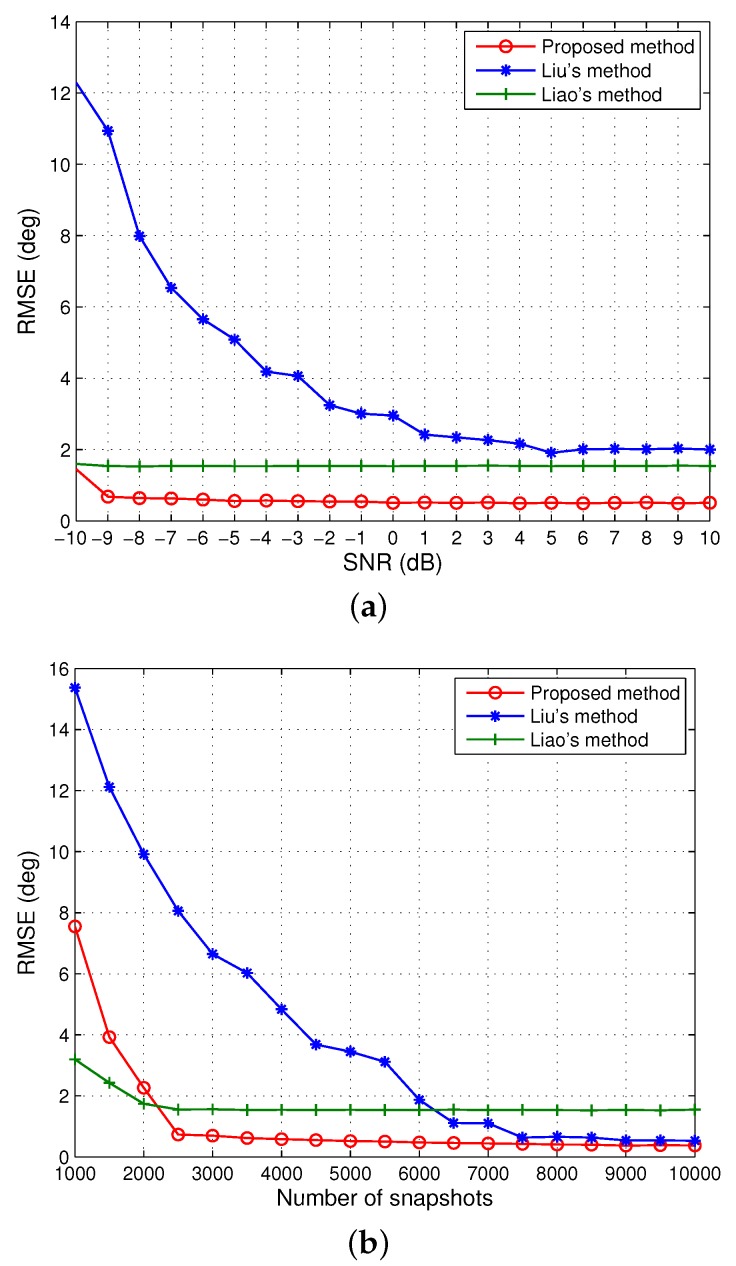
RMSE of the DOA estimates of four signals when M=10 and P=4. (**a**) The number of snapshots is 5000. (**b**) SNR=0 dB.

In the second scenario, we further appraise the performance of the algorithm in this submission under stronger mutual coupling. More precisely, we consider the case of P=4 with $c_{1}=-0.1545+0.4755j,c_{2}=0.1618-0.1176j,c_{3}=0.0211+0.0651j$. The corresponding RMSEs versus SNR and snapshot size are illustrated in Figure 2. It can be seen that the proposed approach performs the best over the entire range of SNR values and the moderate numbers of snapshots. Nevertheless, a performance degradation appears for less than 2000 snapshots mainly because Equation (12) is guaranteed to hold under strong coupling effects only for sufficiently large samples. Contrary to the performance in the first scenario, Liu’s method is strictly inferior to Liao’s method for all SNRs. As analyzed in Section 4.1, the size of the middle sub-array adopted by Liu’s method is M−2P+2=4, and the size of the noise subspace is 7×3, which is much smaller than the 200×196 noise subspace of the proposed method and the 24×4 signal subspace of Liao’s method. Due to the fact that a consistent estimate of a small subspace requires a sufficiently large number of snapshots, Liu’s method has relatively large errors for a moderate number of snapshots, even at high SNRs.

**Figure 3:**
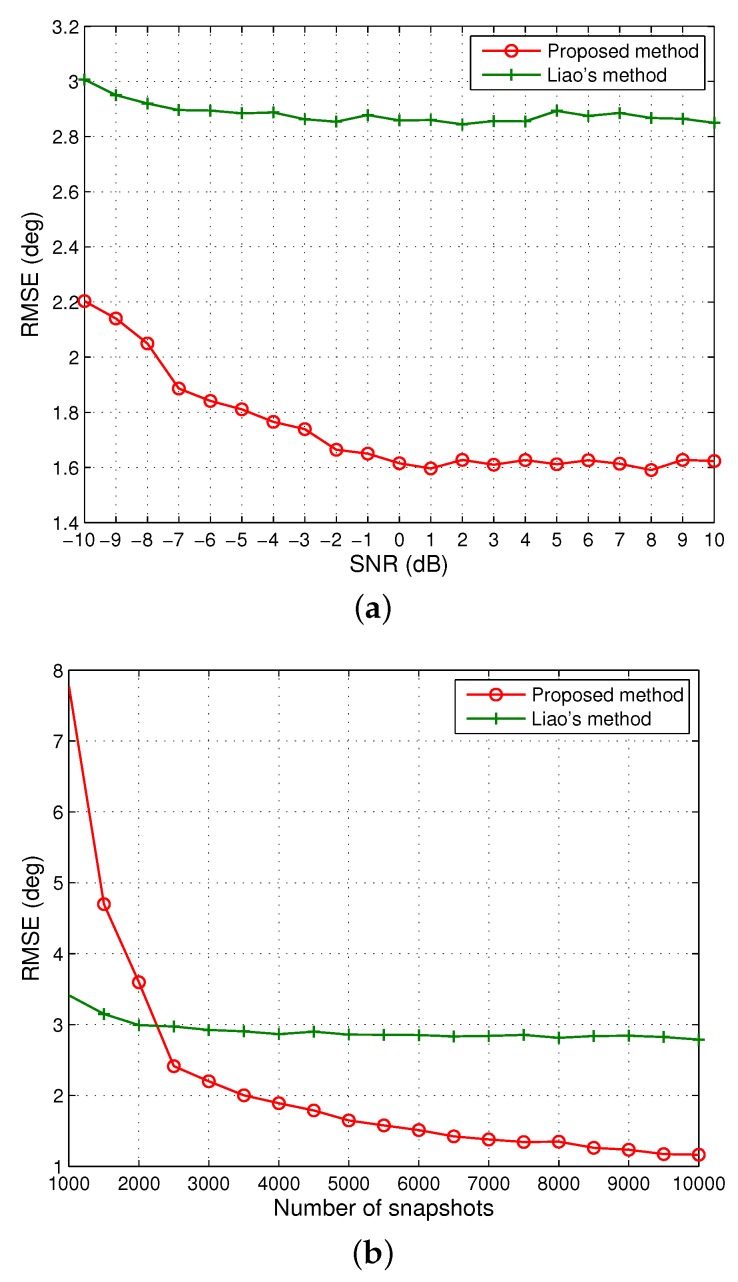
RMSE of the DOA estimates of four signals when M=8 and P=4. (**a**) The number of snapshots is 5000. (**b**) SNR=0 dB.

Next, we change the number of sensors to M=8. The other settings are kept the same as in the second scenario. As discussed in Section 4.1, Liu’s approach fails to work in this example as the number of sensors in the middle sub-array becomes M−2P+2=2, and up to two DOAs can be identified, but actually N=4 signals should be estimated herein. However, the developed algorithm in this submission and Liao’s method can still work under this more challenging scenario. The resultant RMSEs versus SNR and the number of snapshots are depicted in Figure 3. One can observe that the proposed solution significantly outperforms Liao’s method. This can be mainly attributed to the fact that Liao’s method can only utilize the signal subspace with a size of 8×4, whereas the proposed method exploits the noise subspace with a larger size of 128×124 and achieves a lower RMSE for the DOA estimates. However, as in the second scenario, the performance of our method deteriorates quickly and becomes inferior to Liao’s method when the number of snapshots is low. It has been shown in the literature that ESPRIT-like algorithms have clear advantages over MUSIC-like algorithms in terms of estimation errors and robustness to array manifold perturbations in the limited snapshots situation [68]. In our simulations, it was found that at least 2000 snapshots is required to sufficiently characterize the properties of FOC, especially when mutual coupling is relatively strong. It is thus reasonable that Liao’s method (ESPRIT-like) offers better performance than the proposed method (MUSIC-like) when the number of snapshots is lower than 2000.

**Figure 4:**
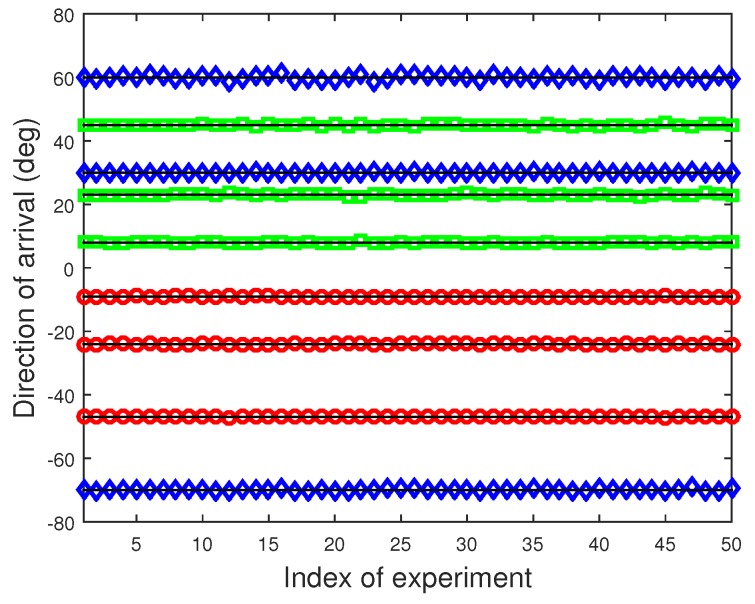
DOA estimates of 50 independent trials using the proposed method, M=10, P=2, SNR=15 dB, and 5000 snapshots.

Then, we verify the proposed scheme for the more challenging scenario where coherency between signals and mutual coupling between sensors coexist. Consider that three groups of coherent signals, three paths per group, from $\left[-47^{\circ },-24^{\circ },-9^{\circ }\right]$, $\left[-70^{\circ },30^{\circ },60^{\circ }\right]$, and $\left[8^{\circ },23^{\circ },45^{\circ }\right]$ impinging on a ten-element coupled ULA with P=2 and $c_{1}=-0.1545+0.4755j$. The fading amplitudes of the coherent signals were $\left[1,0.9,0.8\right]$, $\left[1,0.7,0.6\right]$, and $\left[1,0.7,0.4\right]$, while the fading phases were $\left[48.74^{\circ },121.15^{\circ },35.66^{\circ }\right]$, $\left[9.35^{\circ },251.47^{\circ },103.56^{\circ }\right]$, and $\left[130.21^{\circ },16.88^{\circ },319.69^{\circ }\right]$, respectively. The number of times of FBSS is q=2, and the SNR and number of snapshots are fixed at 15 dB and 5000. Figure 4 illustrates the DOA estimates from 50 independent trials by the developed method in such a harsh case. The solid lines mark the true DOAs. It can be seen that each DOA is correctly determined, and our solution performs satisfactorily.

**Figure 5:**
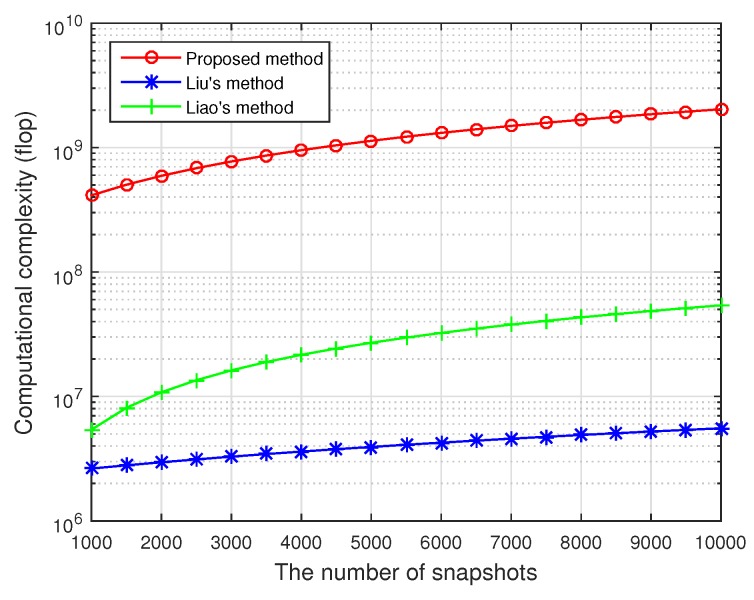
Computational complexity versus the number of snapshots, M=10, P=3, and N=2.

In the last experiment, the computational complexity of the three algorithms versus the number of snapshots is examined. The searching step-size in the MUSIC-like methods is set as $0.1^{\circ }$, and the number of snapshots varies from 1000 to 10,000. Suppose that the number of sensors in the ULA M=10, the coupling length P=3, and the number of independent signals N=2. It can be observed from Figure 5 that our method costs the highest computation, followed by Liao’s method, while Liu’s method is most computationally efficient, which supports the analysis in Section 4.2.

## 7. Conclusions

This paper has addressed the problem of DOA estimation of non-circular signals with ULAs under the coexistence of unknown mutual coupling and colored noise. A new MUSIC-like approach in the context of FOC is developed to offer superior performance over the existing techniques. The proposed algorithm makes efficient use of the extended array observations and enables us to deal with DOA estimation without *a priori* knowledge of mutual coupling between sensors. In addition, a solution to coherent signals with mutual coupling between sensors is introduced, by the middle sub-array to suppress the electromagnetic nuisance and blind separation of coherent signals to enhance DOFs. Compared with the previous work, our solution is able to handle more signals and achieve more accurate DOA estimates even with relatively stronger mutual coupling. A series of simulation results verify the validity and efficiency of the proposed method.
